# Intraoperative Doppler flowmetry evaluation of humeral head perfusion after proximal humerus fracture

**DOI:** 10.1016/j.jseint.2024.06.012

**Published:** 2024-07-08

**Authors:** Doruk Akgün, Alp Paksoy, Jan-Philipp Imiolczyk, Soraya Bahlawane, Henry Gebauer, Rony-Orijit Dey Hazra, Ulrich Stöckle, Karl Friedrich Braun, Philipp Moroder

**Affiliations:** a Charité University Hospital, Center for Musculoskeletal Surgery, Berlin, Germany; b University Hospital rechts der Isar, Technical University Munich, Germany; c Orthopaedic clinic, shoulder and elbow surgery, Schulthess Klinik, Zurich, Switzerland

**Keywords:** Proximal humerus fracture, Post-traumatic osteonecrosis, Doppler sonography, Doppler flowmetry, Pulse-synchronous perfusion, Avascular necrosis

## Abstract

**Background:**

Understanding vascularity and assessing the risk of post-traumatic avascular necrosis are crucial for predicting outcomes and identifying optimal treatment options in proximal humerus fractures (PHFs). Until now, Hertel et al have been the only researchers to evaluate the intraoperative perfusion of the humeral head after fracture using Doppler flowmetry in a central single drill hole within the head. This pilot study aims to standardize the evaluation of intraoperative perfusion measurements in different areas of the humeral head in patients with PHF.

**Methods:**

In this prospective pilot study, intraoperative semiquantitative Doppler perfusion measurements were conducted during plate osteosynthesis for PHF treatment in our institution between July 2021 and May 2022. The fracture morphology was classified radiologically according to Resch's criteria. Quality of reduction was determined postoperatively to be either anatomical, minor malreduced, or major malreduced according to Peters et al in conventional and computed tomography examinations. Medial hinge integrity and medial metaphyseal extension were assessed radiographically according to Hertel et al. Intraoperatively, after drilling screw holes through the plate, a Doppler probe was inserted through all nine drill holes on the humeral head and at least one on the humeral shaft to successively measure the presence of a pulse to indicate if perfusion is present.

**Results:**

A total of ten patients (mean age 59 years, range, 36–83) with a humeral head fracture (2 × 2GL, 3 × 3G, 2 × 4G, 2 × 4GL, 1 × 5aG according to Resch) were included. Nine of the ten patients showed a pulse signal on the humeral shaft. Overall, pulse-synchronous perfusion was detected using Doppler sonography in at least one hole in the humeral head of all patients. In patients with an intact medial hinge (N = 6), pulse-synchronous perfusion could be measured in almost twice as many humeral head holes on average (5.7 vs. 3.0 drill holes) compared to patients with a dislocated medial hinge (N = 4). In patients with metaphyseal extension (N = 3), pulse-synchronous perfusion was measured in an average of 6.7 humeral head holes compared to 3.7 holes in patients without metaphyseal extension (N = 7).

**Conclusion:**

Semiquantitative, intraoperative Doppler flowmetry offers a noninvasive and rapid assessment of humeral perfusion which allows an understanding of humeral head perfusion, when used in a standardized fashion to measure flow in different areas of the humeral head.

Proximal humerus fractures (PHFs) are the third most common type of fracture.^21^ Their incidence is rising due to an aging population and estimated to triple by 2030.^10^^,^^16^ Treatment options range from conservative approach with immobilization to head-preserving reconstruction or prosthetic joint replacement. Despite the frequency of PHFs, the optimal treatment algorithm remains elusive due to a lack of valid scientific evidence.^13^ A comprehensive survey across 743 hospitals in Germany, Austria, and Switzerland showed a current trend of favoring stabilization with fixed-angle implants over arthroplasty in managing these fractures.^22^ The outcome of this preferred treatment option, however, is limited by the blood supply of the humeral head and the associated risk of avascular necrosis (AVN). A deeper understanding of fracture-related ischemia in the humeral head and its correlation with AVN is a key parameter regarding the choice of the most appropriate surgical treatment modality of PHF, osteosynthesis, or arthroplasty. Hertel et al^9^ analyzed the potential risk factors for ischemia of the humeral head postfracture. The assessment involved drilling a central hole in the humeral head to detect the backflow of blood, supplemented by laser Doppler flowmetry in 46 out of 100 patients. The authors identified posteromedial metaphyseal head extension of less than 8 mm, disruption of the medial hinge, and fracture patterns involving the anatomic neck as predictors of ischemia.^9^ The combination of all three factors led to a 97% positive predictive value for the development of ischemia. However, initial ischemia did not significantly alter the rate of AVN in those patients. Some patients with initially intact perfusion of the humeral head later developed AVN and vice versa, suggesting the potential occurrence of revascularization. However, the reasons for late necrosis in initially perfused heads remain unclear.

It is essential to understand perfusion of the humeral head in patients with PHFs in order to understand the risk of developing AVN. This could improve treatment algorithms and decrease the rate of AVN by improving patient selection for reconstruction versus arthroplasty. The purpose of the present pilot study was to evaluate the feasibility of an intraoperative perfusion measurement of the humeral head in a standardized manner using semiquantitative, intraoperative Doppler flowmetry of various head zones in patients with PHF.

## Materials and Methods

### Study design and cohort

The study protocol was reviewed and approved by the institutional ethics committee prior to commencement (EA1/105/20). Ten patients (aged > 18 years) with PHFs who required open reduction and plate osteosynthesis for definitive treatment between July 2021 and May 2022 at our institution were included in the present pilot study. Written informed consent was obtained from all patients prior to inclusion. Exclusion criteria included PHFs older than seven days; isolated tuberosity fractures; diaphyseal extension; pathological fractures; bilateral synchronous fractures; previous ipsilateral shoulder injuries; multiple injuries and integumental or associated vascular injuries.

### Evaluation and description of fracture

All patients underwent biplane radiography, including anteroposterior and transscapular views, along with computed tomography imaging. Axial, oblique coronal, and oblique sagittal images adapted to the shoulder's plane were generated. The image sets of all patients were then analyzed by three shoulder surgeons (D.A., A.P., J.P.) based on a previously published questionnaire by Resch et al^19^ and the fractures were classified using this classification system. Moreover, the quality of reduction was radiographically determined postoperatively by the same surgeons to be either anatomical; minor malreduction; or major malreduction, according to Peters et al.^17^ Furthermore, medial hinge displacement (<2 or ≥2 mm) and medial metaphyseal extension (<8 or ≥8 mm) were assessed following Hertel et al's^9^ criteria in conventional and computed tomography examinations.

### Surgical treatment and implant design

All surgeries followed a standardized protocol at our department.^11^ The patients were positioned in a beach-chair with general anesthesia combined with interscalene block. Following a deltopectoral approach, traction sutures through the rotator cuff or/and Kirschner wires are used to control the humeral head and tuberosities. Crucial steps included medial hinge and humeral head reduction, followed by anatomic reduction of the greater and lesser tuberosities. The most important requirements to promote revascularization were the adequacy of reduction and the stability of fixation while avoiding additional iatrogenic devascularization of the tuberosities. For osteosynthesis, a three-hole 3.5-mm locking proximal humerus plate PHILOS (DePuy Synthes, Raynham, MA, USA) was used in every case, positioned 8 mm distal to the rotator cuff insertion and centered on the greater tuberosity, as recommended by the manufacturer. Screws were then placed into the shaft and head to stabilize the plate. Additional small holes at the proximal part of the plate were used for the fixation of sutures, which were applied into the rotator cuff tendons to neutralize muscle forces.

### Evaluation of perfusion

**Figure 1:**
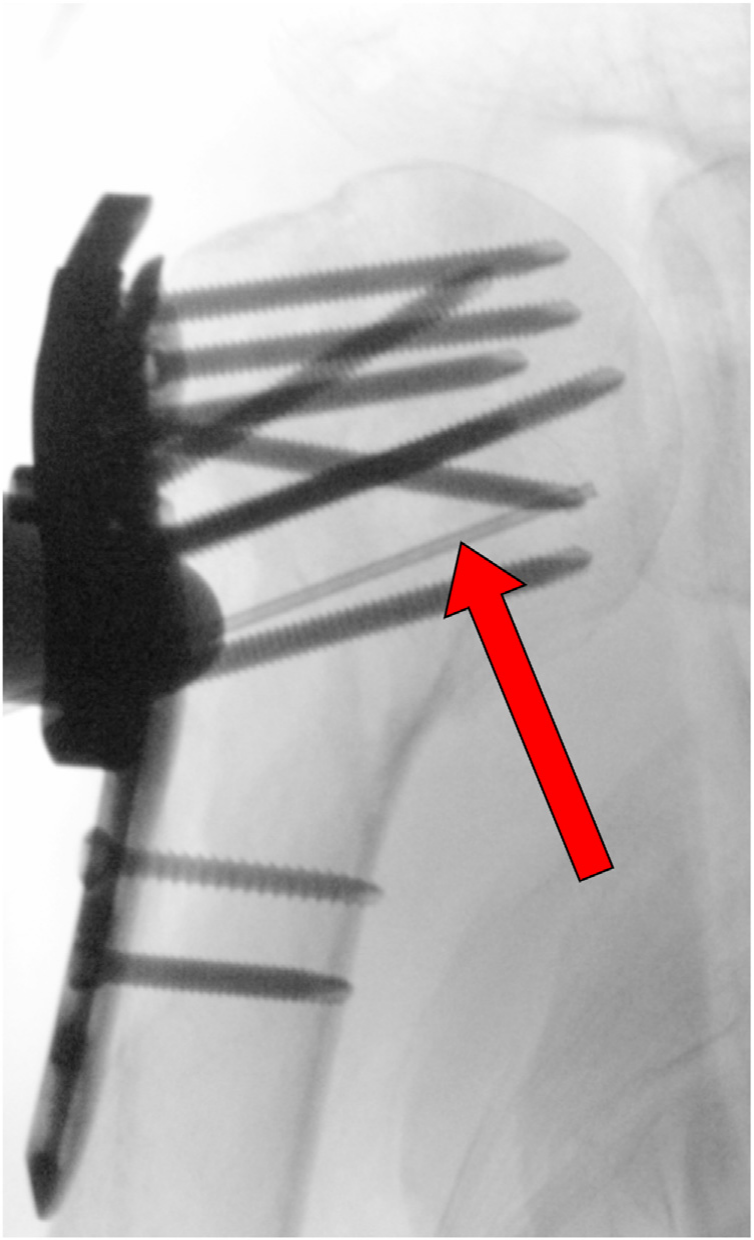
Intraoperative fluoroscopy view illustrating anteroposteriorly the Doppler probe inserted into the cancellous bone through the posterior E hole of the plate postdrilling.

**Figure 2:**
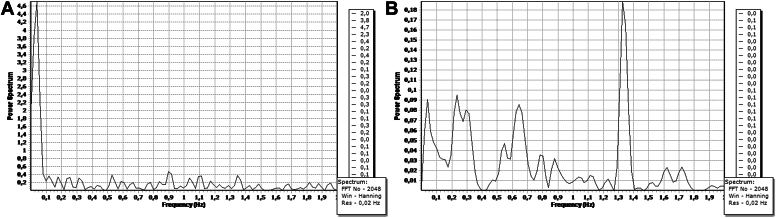
Examples of perfusion measurements in drill holes of a female patient with an approximate heart rate of 100/min. (**A**) No significantly outlying amplitude height can be seen between 1.3 and 1.4 Hz, indicating no existing perfusion in the drill hole measured. (**B**) A significantly outlying amplitude height can be seen between 1.3 and 1.4 Hz, serving as proof of perfusion in the drill hole measured.

In order to measure bony perfusion, a new laser Doppler flowmetry source was designed specifically (Moor Instruments, Axminster, UK) and used in combination with a conventional laser Doppler flowmeter (DRT4; Moor Instruments). The source energy was 20 mW (emitting laser light with a wavelength of 780 nm). Perfusion of the fractured humeral head was evaluated intraoperatively by laser Doppler flowmetry measurements after insertion of the Doppler probe into the cancellous bone through the previous drill holes of the plate postdrilling (Fig. 1). In each case, different zones of the humeral head were independently examined by Doppler flowmetry for perfusion through predefined drill holes in the plate. With the probe in position, the Doppler flux measurement settled until a sinusoidal wave pattern synchronized with the pulse rate was obtained. The data were continuously recorded in real-time mode for 30 seconds and stored for later analysis using the DRT4 Windows computer software program. Blood flow was measured in flux units (Perfusion units) which are arbitrary units related to mean red blood cell flow. These were defined by calibration against a standard reference of polystyrene microspheres, as provided by the manufacturer (Moor Instruments). The measured amplitude height depended on the specific probe position. Relative changes in amplitude identified maneuvers that altered perfusion. The time constant was set at 0.1 seconds, and the display rate was set at 60 per second to enable the visualization of pulsatile signals. A noise-free period of 30 seconds was chosen for a stable and reliable recording session. A pulsatile, electrocardiographic synchronous signal served as proof of existing perfusion when it showed the same frequency as the pulse of the patient (Fig. 2).

### Rehabilitation protocol

Patients adhered to a standardized postoperative rehabilitation protocol. The injured shoulder was immobilized in a sling in internal rotation for two weeks. During this time, only passive pendulum movement (excluding external rotation above 0°) was allowed during physiotherapy sessions. After one week, patients actively performed simple pendulum exercises several times a day, assisted external rotation up to 10° less than the opposite side, assisted internal rotation up to the abdomen, and assisted elevation of the hand to the forehead, for five weeks. Active range of motion (ROM) exercises, along with strengthening and resistance training, were carefully introduced after six weeks postoperatively upon radiographic evidence of fracture healing.

### Postoperative follow-up

Clinical and radiological examinations were performed six, 12, and 24 weeks postoperatively or in case of any complaints. Clinical outcomes were documented during follow-up examination using the Constant-Murley Score (CS)^5^ and the patient-reported Subjective Shoulder Value (SSV).^7^ SSV was determined by asking the question, ‘‘Assuming that a normal shoulder scores 100 points, how many points does your shoulder score today?’’. Active ROM was documented for active abduction, active forward flexion, active external rotation (AER), and active internal rotation (AIR). AER was assessed with the patient standing with the arm at their side and AIR as the ability to reach different levels of the spine with the thumb.

### Statistical analysis

The mean, median, standard deviation, and absolute and percentage frequency were calculated for the descriptive analysis. Associations between the number of drill holes with measured pulse-synchronous perfusion and the clinical outcomes CS, SSV, and ROM were assessed using Pearson correlation coefficients. IBM SPSS Statistics 29.0 software (IBM, Armonk, NY, USA) was employed for this analysis.

## Results

A total of ten patients (seven female, three male) with PHF were recruited for the present study. The mean age of all patients at the time of the operation was 59.0 ± 15.6 years. According to Resch et al,^19^ there were two 2GL, three 3G, two 4G, two 4GL fractures, and one 5aG fracture. Out of ten patients, nine had a pulse signal in the humeral shaft. Pulse-synchronous perfusion was detected using Doppler sonography in at least one hole in the humeral head of each patient. In patients with an intact medial cortex (N = 6), pulse-synchronous perfusion was measured on average in almost twice as many humeral head holes (5.7 vs. 3.0 drill holes) compared to patients with a dislocated medial hinge (N = 4). In patients with metaphyseal extension (N = 3), pulse-synchronous perfusion could be measured in an average of 6.7 humeral head holes compared to 3.7 holes in patients without metaphyseal extension (N = 7). In patients with anatomical reduction (N = 7), pulse-synchronous perfusion was measured in an average of four humeral head holes compared to six holes in patients with minor malreduction (N = 3). All demographic details of the cohort and their perfusion measurements with types of fractures are shown in Table 1.

**Table I:** Detailed illustration of each patient included showing baseline demographics, fracture classification according to Resch, radiological reduction analysis according to Peters, perfusion measurements in each hole, medial hinge condition (disrupted/intact), and medial extension existence ("+" meaning presence and "−" meaning absence).

Sex	Side (r/l)	Age (y)	Fracture type	Reduction analysis	A ant/post		B ant/post		C ant/post		D	E ant/post		F (shaft proximal)	G (shaft center)	H (shaft distal)	Medial hinge	Medial extension
Male	r	76	3G	Anatomical	-	-	-	-	+	-	-	-	-	+	+	(−)	Disrupted	-
Female	r	51	3G	Anatomical	+	-	+	+	+	-	+	-	-	+	+	+	Intact	-
Female	l	37	4GL	Minor malreduction	+	+	-	-	+	+	+	+	+	+	-	+	Disrupted	-
Female	l	36	3G	Anatomical	-	-	+	-	-	-	-	-	+	+	-	+	Intact	-
Female	r	69	5aG	Anatomical	+	+	+	+	+	-	+	+	-	+	+	+	Intact	-
Male	l	57	2GL	Minor malreduction	-	-	-	-	-	+	-	-	+	(−)	-	(−)	Disrupted	-
Female	r	83	4G	Anatomical	+	+	-	-	-	-	-	-	-	+	+	(−)	Disrupted	-
Female	r	67	2GL	Anatomical	-	+	+	+	+	-	+	+	+	+	+	(−)	Intact	+
Male	l	63	4GL	Anatomical	-	+	-	+	-	-	+	+	-	+	+	+	Intact	+
Female	r	51	4G	Minor malreduciton	+	+	+	+	+	+	+	+	+	-	+	+	Intact	+

"+" means a pulsatile, electrocardiographic synchronous signal as proof of positive perfusion, "-" no pulse-synchronous perfusion detected, and "(−)" no perfusion measurement conducted in the drill hole.

CS, SSV, ROM, and AVN could be assessed in seven out of ten patients eligible for the final follow-up after a mean period of 1.8 ± 0.4 years. Overall, the mean CS was 73.0 ± 10.0 (range 57–84), while the mean SSV was 74.0 ± 14.6 (range 50–98). The mean active forward flexion was 155.7 ± 32.1° (range 90–180°), the mean active abduction was 138.6 ± 46.0° (range 80–180°), the mean AER was 34.3 ± 14.0° (range 10–30°) and the mean AIR was approximately between the 12^th^ thoracic vertebral body and the shoulder blades (8.6 ± 1.0°). AVN was not observed in any of the patients. No relevant intraoperative complications, neurovascular complications, or infections occurred. No significant correlation could be found between the number of drill holes with measured pulse-synchronous perfusion and the clinical outcomes CS, SSV, and ROM.

## Discussion

The operative treatment options available for patients with three- and four-part PHFs include osteosynthesis or arthroplasty, depending on various factors such as age, daily activity, and fracture pattern.^2^^,^^8^ Adequately managing these complex fractures requires a better understanding of the initial ischemia of the humeral head depending on the fracture pattern and its effect on developing AVN. Preservation of the humeral head is a viable treatment option when adequate reduction and stable conditions for revascularization can be achieved, even in the case of acute ischemia.^9^

In the present study, we performed a standardized measurement of humeral head perfusion through different drill holes and observed pulse-synchronous perfusion using Doppler flowmetry, which appears to be a reliable method for measurement of blood flow in different parts of the humeral head. Residual perfusion of the head appears to rely on the integrity of the medial hinge (of the capsule-periosteum) and metaphyseal extension (the length of the humeral calcar which remained attached to the head). Both reflect the risk of disruption of the proximal anterior and posterior circumflex humeral arteries. The periosteum provides both the mechanical support and biological resources vital for normal bone homeostasis and modeling.^4^^,^^6^ Periosteal bridges between the head fragment and the shaft can preserve the blood supply of the head in PHF.^1^^,^^20^ As already described in the literature, disruption of the medial hinge appears to be a critical factor in displacement-induced stripping of the periosteum (and endosteal vessels).^12^^,^^18^ The integrity of the medial hinge is an important aid in fracture reduction and stabilization, and it determines the potential difficulty of reduction and internal fixation.^9^ As shown in the literature, perfused heads have a significantly longer medial metaphyseal extension than ischemic heads.^9^ This study supports these findings by depicting pulse-synchronous perfusion with Doppler sonography measurements in almost twice as many humeral head holes in patients with an intact medial hinge or metaphyseal extension compared to patients with a disrupted medial hinge or without metaphyseal extension. However, a larger cohort of patients is needed to confirm these findings with statistical significance.

In a study by Hertel et al,^9^ humeral head perfusion was evaluated by observing backflow and by Doppler flowmetry after drilling a central hole. When there was uncertainty (in four cases, where there was a pulsatile Laser signal but the observed bleeding was minimal), they applied up to three drill holes .^9^ The strength of the present pilot study lies in its assessing perfusion intraoperatively in different zones of the humeral head. Different areas of the humeral head were separately examined by Doppler flowmetry through predefined drill holes in the plate to provide a more comprehensive understanding of humeral head perfusion according to the fracture morphology compared to Hertel et al's study, where only the central area of the humeral head could be evaluated. The vascular supply to the humeral head is complex and it is not definitively known how actual humeral head viability correlates with fracture pattern and displacement in PHF.^3^ In order to understand the correlation between perfusion areas and fracture morphology, a larger sample size is needed, which could be pursued as a continuation of this pilot study.

With the use of locking plates, plate osteosynthesis achieves excellent fracture reduction, allows early functional exercise, and results in satisfactory clinical outcomes,^14^^,^^23^ especially in patients below 65  years of age.^15^ In all patients in the study cohort, the last follow-up with biplane radiography confirmed that the fractures had healed well. In the seven patients with follow-up, good-excellent CS, SSV, and ROM were achieved, and their mean age was 60.9 ± 14.9 years with only one patient aged over 70 year old. In agreement with the literature, the present study shows that osteosynthesis with preservation of the humeral head is worth considering in the treatment of complex PHF in young patients.

This study has several limitations. Firstly, the evaluation method of intraoperative perfusion with Doppler flowmetry is a semiquantitative measurement. Positive pulse-synchronous perfusion indicates intact perfusion, while lack of signal indicates neovascularized bone. However, the measurements between the drill holes cannot be evaluated in a comparative manner as the magnitude of the pulse-synchronous perfusion signals do not correlate with the absolute perfusion amount in the drill hole measured. Secondly, the sample size is small due to its nature as a pilot study. A larger sample size would have allowed a more powerful statistical subgroup analysis to be performed. The continuation of the present study and further studies with a larger sample size are, therefore, necessary to obtain a more precise efficacy. Lastly, although different surgeons have performed all of the surgeries in a standardized fashion, some differences between individual surgeons with regards to technical influence on the fracture reduction cannot be ruled out.

## Conclusion

Semiquantitative, intraoperative Doppler flowmetry offers a noninvasive and rapid assessment of humeral head perfusion when used in a standardized fashion to measure different humeral head areas.

## Disclaimers:

Funding: The authors disclose receipt of the following financial or material support for the research, authorship, and/or publication of this article: German, Austrian and Swiss Shoulder and Elbow Society.

Conflicts of interest: Doruk Akgün reports that this study was supported by the German, Austrian, and Swiss Shoulder and Elbow Society. All the other authors, their immediate families, and any research foundations with which they are affiliated have not received any financial payments or other benefits from any commercial entity related to the subject of this article.
